# Synthesis, characterization, and anti-cancer activity evaluation of Ba-doped CuS nanostructures synthesized by the co-precipitation method

**DOI:** 10.1039/d4ra07078j

**Published:** 2025-02-12

**Authors:** A. M. Abdulwahab, Asma'a Ahmed AL-Adhreai, A. H. Al-Hammadi, Arwa Al-Adhreai, Aeshah Salem, Faisal Katib Alanazi, Mohammed ALSaeedy

**Affiliations:** a Department of Physics, Faculty of Applied Science, Thamar University Dhamar 87246 Yemen abduhabdulwahab@yahoo.com Asma.AlAhreai@gmail.com; b Department of Physics, Faculty of Science, Sana'a University Sana'a Yemen; c Department of Chemistry, Faculty of Applied Science, Thamar University Dhamar 87246 Yemen; d Department of Physics, Faculty of Science, Taibah University Yanbu 46423 Saudi Arabia; e Department of Physics, College of Science, Northern Border University Arar 73222 Saudi Arabia; f Department of Chemistry, Faculty of Education & Science, AlBaydha University Al-Baydha Yemen

## Abstract

Pure and barium (Ba)-doped copper sulfide nanostructures were synthesized by the chemical co-precipitation method at room temperature. The pure and Ba-doped CuS nanoparticles have been compared structurally, morphologically, and optically using X-ray diffraction (XRD), transmission electron microscopy (TEM), total reflection X-ray fluorescence (TXRF), diffuse reflectance spectroscopy (DRS) and photoluminescence (PL) spectroscopy. The results of X-ray diffraction (XRD) showed that the CuS nanostructures have a hexagonal structure with crystallite sizes ranging from 15.14 to 16.69 nm. TXRF was used to confirm the presence of Ba-doped CuS. Diffuse reflectance spectroscopy (DRS) analysis revealed that the bandgap energy of the CuS nanostructures increased with increasing Ba doping concentration (1.38 to 1.82 eV). Optical constants such as absorption coefficient, extinction coefficient, and refractive index were calculated. A photoluminescence study of CuS was carried out. One photoluminescence (PL) band was found at 826 nm (1.5 eV) at room temperature and was attributed to band-to-band (BB) and band-to-impurity recombination. The MTT assay was used to measure the cytotoxicity effect on human lung cancer cell lines (A549). The results showed that CuS nanostructure with 5% Ba doping exhibits more toxicity than other samples, with an IC_50_ of 8.46 g ml^−1^ being the most significant concentration, suggesting that it may be a promising cancer treatment agent.

## Abstract 2

Synthesis, characterization, and anti-cancer potential of Ba-doped CuS nanoparticles.

## Graphical abstract

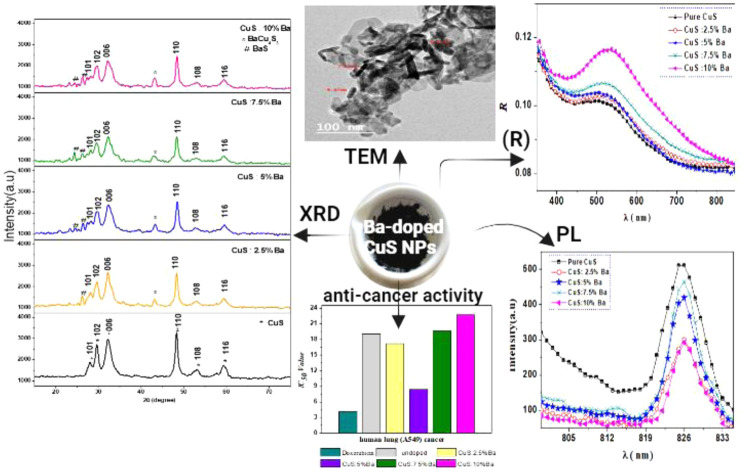

## 1. Introduction

The unique chemical and physical properties of transition metal chalcogenide compounds, which depend on the morphology of the nanomaterials, have recently attracted a lot of study interest.^1^ A lot of research on metal sulfides semiconductors focuses on chalcogenides (group 12), particularly zinc and cadmium sulfide. However, the toxicity of these materials limits their potential uses, which is why copper sulfide nanocrystals have recently attracted attention for a variety of uses.^2^ CuS is more advantageous than other materials due to its nontoxic, abundant elemental composition, low cost, electronic conductivity, and chemical stability at ambient temperatures.^3^ Numerous studies have been carried out on copper sulfides because of their interesting optical, chemical, biological, and electrical properties, as well as their great potential in a variety of applications, including batteries, solar cells, cancer therapy, nonlinear optical materials, and heterogeneous catalysis.^4^ The optical band gap energy of CuS nanoparticles (NPs) varies depending on stoichiometry and is between 1.2 and 2.5 eV. Numerous studies have been carried out on copper sulfide nanoparticles (NPs) with various crystalline phases, including chalcocite (Cu_2_S), djurleite (Cu_1.9_S), digenite (Cu_1.8_S), anilite (Cu_1.75_S), and covellite (CuS).^5^ Researchers are particularly interested in the greenish-black solid covellite CuS because it possesses a conductivity similar to metal, can sense chemicals, and is capable of becoming a superconductor at a low temperature of 1.6 K.^6^

Doping can successfully regulate phase composition and microstructure. Doping incorporation has enhanced the dielectric, morphological, electric, catalytic, and optical properties of CuS NPs. Several doping elements like vanadium, cobalt, gallium, indium, silver, and nickel have been reported as dopants in CuS.^7^ Ba is an element of the family of alkaline earth metals and consistently shows an oxidation state of +2. Ba is useful in a wide range of products, including guiding layers, liquid sensors, ceramics, fluorescent lighting, rubber, and optical glass. The ionic radius for the Ba^2+^ ion is 1.35 Å, while that for the Cu^2+^ ion is 0.73 Å.^8^ Barium (Ba) was used as a dopant in previous research to modify the characteristics of many semiconductors, including CdS,^9^ ZnS,^10^ CdO,^11^ and TiO_2_,^12^ for a number of applications. Therefore, the optical, structural, and electrical properties of the CuS nanoparticles can be significantly affected by the introduction of Ba into the CuS lattice.

To the best of our knowledge, only a few studies have been conducted on the synthesis and characterization of CuS doped with Ba. Many different techniques have been used to synthesize CuS nanoparticles, including chemical co-precipitation (CCP),^13^ sonochemical synthesis,^14^ the sol–gel method,^15^ hydrothermal,^16^ and irradiation techniques.^17^ The chemical co-precipitation method (CCP) stands out among these synthesis procedures since few of the others have required high temperatures, hazardous solvents, or several steps in the synthesis process. These additional benefits of the co-precipitation approach simple and inexpensive technology, easy control over particle size, and a lower temperature requirement (90 °C)—are all advantages of this method.^18^ The aim and novelty of this work is to report the effects of doping CuS with Ba on the structural and optical properties of the host CuS. Also, to the best of our knowledge, there is no previous study about the effect of Ba-doped CuS on the lung cancer cell, so CuS nanostructures will be biologically studied by examining the anticancer toxicity against human lung (A549) cancer cells using the MTT assay.

## 2. Materials and method

### 2.1 Materials

Copper chloride 2-hydrate (CuCl_2_·2H_2_O, ≥99%), barium chloride dehydrates (BaCl_2_·2H_2_O, ≥99%), and sodium sulfide hydrate (Na_2_S·*x*H_2_O, ≥65%). All of the chemicals in this work have been used without further purification. Distilled water was used for preparing all of the aqueous solutions.

### 2.2 Preparation of pure and Ba doped CuS nanoparticles

Copper chloride 2-hydrate and sodium sulfide hydrate were employed as source precursors for the synthesis of CuS nanoparticle material using the co-precipitation method, which allowed for the growth of a pure CuS sample.

**Fig. 1:**
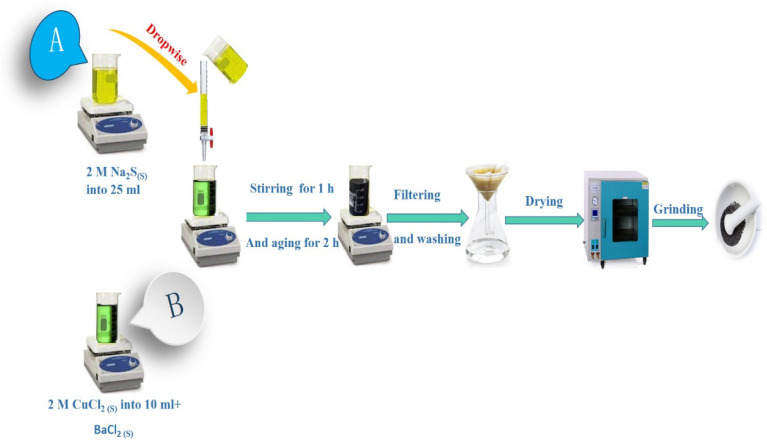
Schematic diagram of the steps used for the synthesis of pure and Ba-doped CuS nanoparticles.

In order to accomplish total dissolution in solution (A), 2 M of sodium sulfide hydrate was dissolved in 25 ml of distilled water and continuously stirred for 20 minutes at room temperature. In solution (B), 10 ml of distilled water was mixed with 2 M copper chloride 2-hydrate for 20 minutes. Drops of the homogenous solution (A) were added to the solution (B), which was then stirred using a magnetic stirrer for 1 hour at room temperature. The produced solution was aged for two hours, and the end product was filtered through filter paper and washed many times with distilled water and ethanol in order to remove impurities. To obtain the dark greenish powder sample, it was then dried for three hours at 100 °C in the oven. Before further characterization, it is gently ground in a mortar to produce the material in powder form. Barium chloride dehydrate, with *x* = 0.025, 0.05, 0.075, and 0.1, was added to a solution of copper chloride 2-hydrate (solution B), and the same process described above was then used to produce Cu_1−*x*_Ba_*x*_S nanoparticles with various doping concentrations. As shown schematically in Fig. 1, the procedures followed to produce hexagonal copper sulfide nanoparticles.

### 2.3 Characterizations

Various characterization investigations were performed on the obtained samples of nanoparticles. Utilizing an XD-2 X-ray diffractometer with CuK-1 radiation of *λ* = 0.154056 nm, the crystal structure and crystallite size of the materials were studied. The corresponding selected area electron diffraction (SAED) pattern and the high-resolution transmission electron micrograph (HR-TEM) were obtained by Transmission Electron Microscopy (JEM-2100, Japan). ImageJ 1.53e software was used to evaluate the size of TEM images. A total reflection X-ray fluorescence (TXRF) spectrometer (Bruker S8 TIGER) was used to do the elemental analysis. The reflectance spectra transformation was measured using an ultraviolet-visible-near infrared (UV-VIS-NIR) diffuse reflectance spectrophotometer (V-570). The photoluminescence measurements were performed on the spectrofluorometer FS5 (Edinburgh). Origin Pro 8 was used to plot the graphs.

### 2.4 Anticancer activity of CuS nanoparticles

#### 2.4.1 Cell culture

The human lung (A549) cancer cell lines were obtained from American Type Culture Collection (ATCC) *via* Holding company for biological products and vaccines (VACSERA), Cairo, Egypt. Cells were cultured using DMEM (Gibco, USA) supplemented with 10% FBS (Gibco, USA) and a 1% penicillin–streptomycin mixture (100 IU ml^−1^ penicillin and 0.1 mg ml^−1^ streptomycin) at 37°C. Other chemicals and reagents were all purchased from Sigma or Invitrogen.

#### 2.4.2 *In vitro* MTT cell proliferation assay

MTT assay: using the MTT colorimetric cell viability assay, the cytotoxicity of the samples was evaluated against human lung (A549) cancer cell lines. Cells at a density of 2–2.5 ×10^4^ cells per well were cultured in a 96-well tissue culture plate with a volume of 180 μl complete growth medium per well for 24 hours before being treated with 20 μl dilutions of the tested substance. DMSO was used to make drug stock solutions. In the growth medium, eight concentrations (300, 100, 30, 10, 3, 1, 0.3, and 0.1 μg ml^−1^) of each compound were prepared. The cells were then treated for 72 hours.

Each well was then filled with freshly produced MTT salt (5 mg ml^−1^; Sigma). After carefully removing the MTT solution, an equivalent volume of 200 μl DMSO was then added to each well and incubated for 60 minutes while shaking. To detect the growth of cells, the Multiskan® EX (Thermo Scientific, USA) MicroPlate Reader was used to measure the absorbance of each well at 590 nm. The experiment was carried out three times in parallel. IC_50_ values were calculated using GraphPad Prism and a non-linear fit of the dose–response curve.

## 3. Results and discussion

### 3.1 XRD analysis

**Fig. 2:**
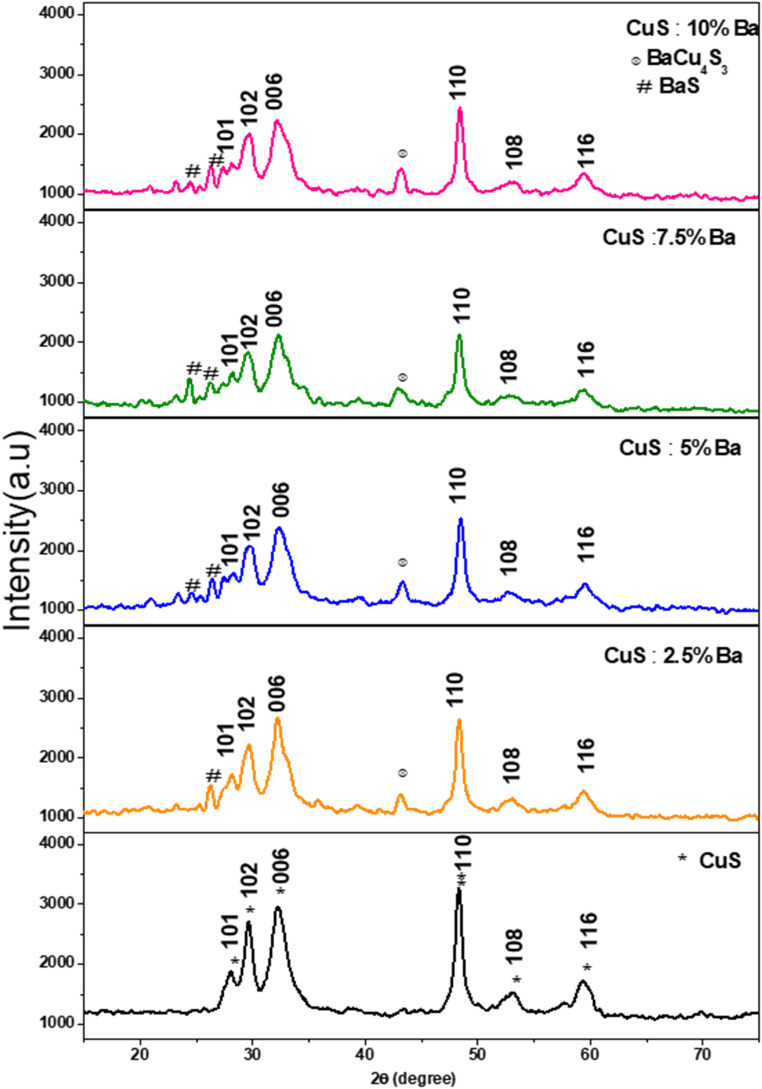
XRD spectra of pure and Ba-doped CuS nanoparticles.

**Fig. 3:**
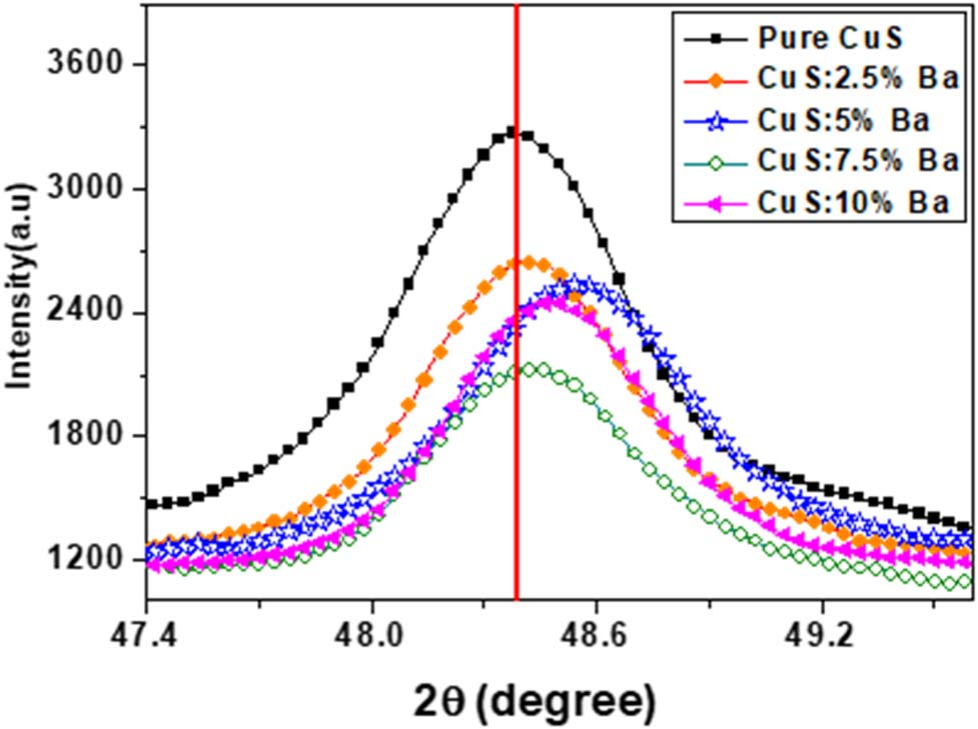
Peak shifting of pure and Ba-doped CuS nanoparticles.

Fig. 2 shows the XRD pattern of CuS and Ba-doped CuS nanostructures at concentrations of 2.5%, 5%, 7.5%, and 10%. The peaks of the (101), (102), (006), (110), (108), and (116) planes in the diffraction spectra represent the hexagonal phase of pure covellite of CuS. The cell characteristics are *a* = 3.792 Å and *c* = 16.344 Å, and all of the measured peaks match the peaks in JCPDS card no. 06-0464. Except for the hexagonal phase, no other phase was seen. However, as Ba dopant concentrations increased, we noticed some new impurity peaks at locations 2*θ* = 24.3° and 43.5°, which demonstrates that Ba is present in the CuS lattice. By comparing their peaks with JCPDS cards, the existence of impurities was confirmed. JCPDS card no. 08-0454 for BaS and JCPDS card no. 50-0690 for BaCu_4_S_3_. It is evident that the (110) plane has a high intensity peak, indicating that nanocrystallites prefer to form on this plane as their preferred crystal plane. Furthermore, Table 1 lists the major peak position and full width at half maxima, clearly showing that Ba doping has little impact on CuS microstructure. As can be observed in Fig. 3, as the barium concentration in the CuS sample increases to 7.5%, the intensity decreases, followed by an increase in its value. The intensities of the peaks, however, significantly decreased when CuS was doped with 7.5 percentages of Ba impurities. Also, the position of the peaks of Ba-doped CuS shifted a little to higher 2*θ* angles. In actuality, the CuS lattice is distorted because of the substitution or interstitial of Ba atoms in the CuS structure.^19^

**Table 1:** XRD data of pure and Ba-doped CuS nanoparticles

Samples	*d* (Å)	(2*θ* degree)	(*β* degree)	*D* (nm)	*a* (Å)	*c* (Å)	*δ* × 10^15^ (m^−2^) dislocation density	*V*_cell_ (Å)^3^
Pure	1.8776	48.38	0.575	15.14	3.755	16.369	4.36	199.912
2.5% Ba	1.8784	48.42	0.535	16.28	3.757	16.224	3.77	198.308
5% Ba	1.8725	48.58	0.562	15.51	3.745	16.331	4.16	198.354
7.5% Ba	1.8791	48.42	0.522	16.69	3.758	16.235	3.59	198.580
10% Ba	1.8769	48.48	0.532	16.37	3.754	16.438	3.73	200.598

Using the Scherer formula, the high intensity peak of the X-ray diffraction patterns was used to determine the crystallite size (*D*) of pure and Ba-doped CuS nanostructures:^20^1
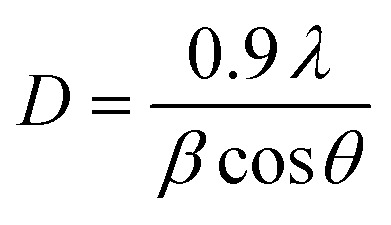
where *β*, *λ*, and *θ* are full width at half maxima, the wavelengths of X-ray radiation, and Bragg's angle. The outcomes show that the size of the crystallites changes irregularly with doping when the dopant percentage is increased. When compared to the doped sample, these values are higher (see Table 1). Higher crystallite sizes (16.69 nm) were seen in CuS that had 7.5% Ba doped. This increase is explained by the fact that Ba^2+^ has a larger ionic radius (1.35 Å) than Cu^2+^ (0.73 Å).

The following equation determines the lattice constants *a* and *c* for a hexagonal-phase structure,^21^ and the obtained values are reported in Table 1.2
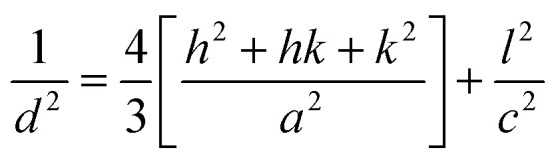


Table 1 displays the changing lattice constants with barium doping concentration on the CuS nanostructure. The following relation describes the dislocation density, which is the length of the dislocation lines in a unit volume of the crystal:^22^3
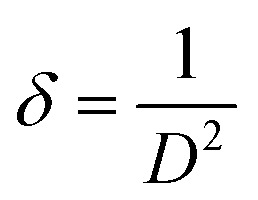
where *D* is the size of the crystallite. The dislocation density, in general, describes the quantity of defects generated in crystalline materials.^3^

The volume of the unit cell of the hexagonal system was calculated. using equation.^23^

4 *V* = 0.866 × *a*^2^ × *C*

The dislocation density and volume of the unit cell in the hexagonal crystal structure are presented in Table 1. As can be seen from Table 1, dislocation density values decrease with Ba doping, leading to improved CuS crystallinity. Ba doping has improved the CuS crystal structure, which explains why the *δ* values have decreased. Additionally, doping until 7.5% of Ba-doping increases and reduces the volume of the unit cell. According to Vegard's law,^24^ doping can take place at interstitial locations, which helps to explain how the volume of the CuS matrix changes when Ba is incorporated into it.

### 3.2 TEM and SAED analysis

**Fig. 4:**
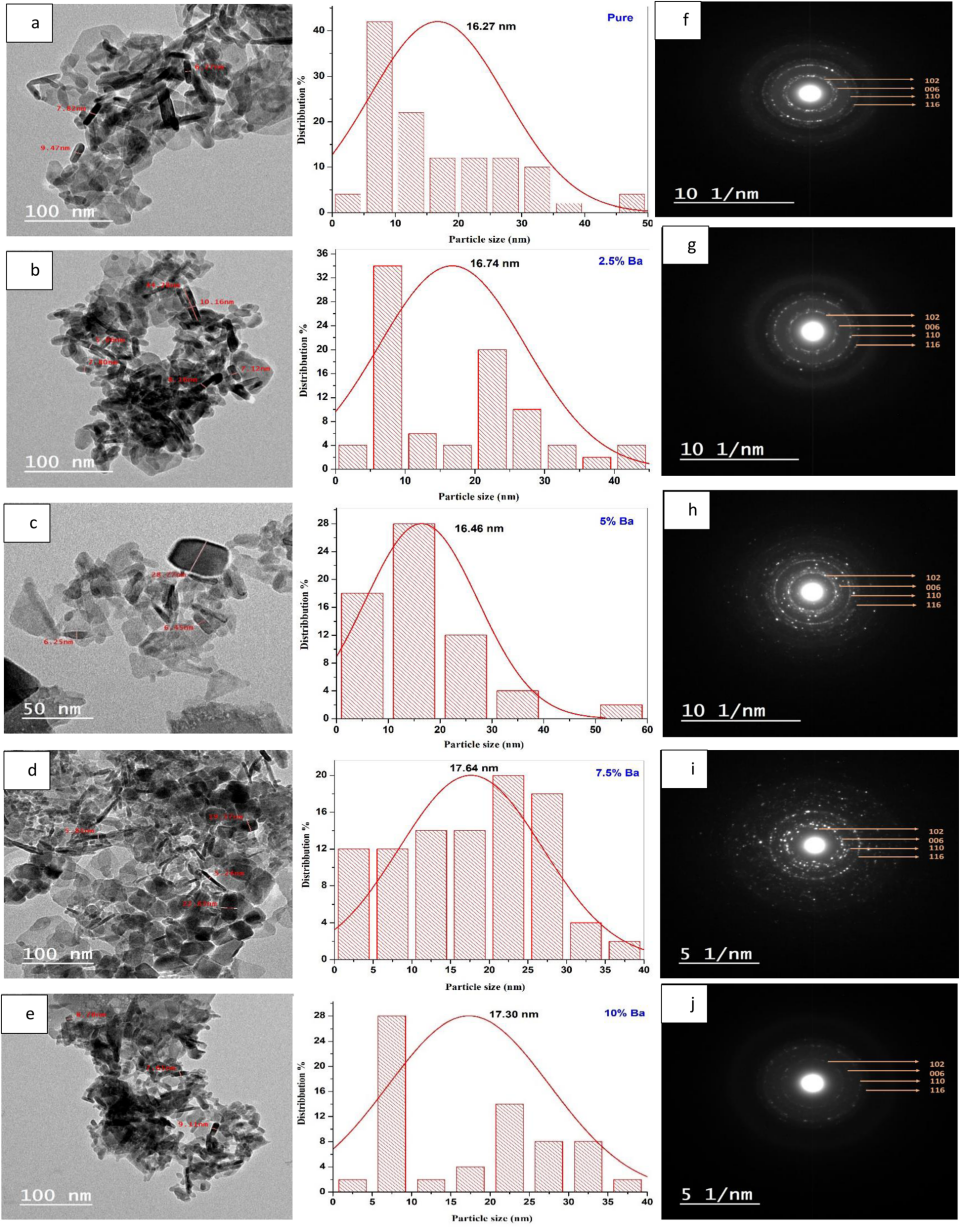
TEM images of Ba-doped CuS nanoparticles with their respective particle size distribution histograms: pure CuS (a) 2.5% Ba (b), 5% Ba (c), 7.5% Ba (d), 10% Ba (e), Selected Area Electron Diffraction (SAED) pattern of Ba-doped CuS nanoparticles: pure CuS (f) 2.5% Ba (g), 5% Ba (h), 7.5% Ba (i), 10% Ba (j).

TEM images of the samples were examined to estimate the size of the produced nanoparticles and define their morphologies. Fig. 4(a–e) presents TEM images of CuS and Ba-doped CuS nanoparticles with their respective particle size distribution histograms. Agglomeration of the particles was observed, which resulted in a variation in the particle size. The morphology of pure CuS consists of nanorods and hexagonal with an average particle size of 16.27 nm, while 2.5% Ba-doped CuS nanoparticles are mostly nanorods with an average size of 16.74 nm. With an average size of 16.46 nm, the 5% Ba-doped CuS consists of a variety of nanoparticle shapes, including triangular, rectangular, and nanorod. Thin nanorods, rectangular, cubic, and irregular shapes (17.64 nm) were obtained with 7.5% Ba-doped CuS nanoparticles. 10% Ba nanoparticles most often have a nanorod shape, but near-spherical, irregular, and other shapes were also found (17.30 nm). The resultant (SAED) pattern of the copper sulfide nanoparticles (Fig. 4(f–j)) revealed the crystalline structure and may be indexed to the hexagonal Ba-doped CuS nanostructures' (102), (006), (110), and (116) planes. The sample's polycrystalline nature is indicated by well-defined diffraction rings in the SAED pattern, which is consistent with the XRD observation.

### 3.3 Total reflection X-ray fluorescence (TXRF)

The quantitative analysis of each sample was determined using TXRF. Table 2 displays the elemental compositions of CuS and Ba-doped CuS nanoparticles. Cu and S are the major components in all samples, while Ba is the minor element. The results confirm the presence of Ba in the CuS nanoparticle structure. Other elements (Cl, P, and Ca) were detected as traces.

**Table 2:** TXRF results of the analyzed samples

Samples	Cu, %	S, %	Ba, %	Cl, P, Ca (traces), %
Pure	55.25	44.39	—	0.36
2.5% Ba	53.40	44.23	2.05	0.32
5% Ba	52.36	43.42	3.9	0.32
7.5% Ba	51.01	42.20	6.45	0.34
10% Ba	51.35	43.53	4.76	0.36

### 3.4 Optical analysis

#### 3.4.1 The diffused reflectance (*R*)

**Fig. 5:**
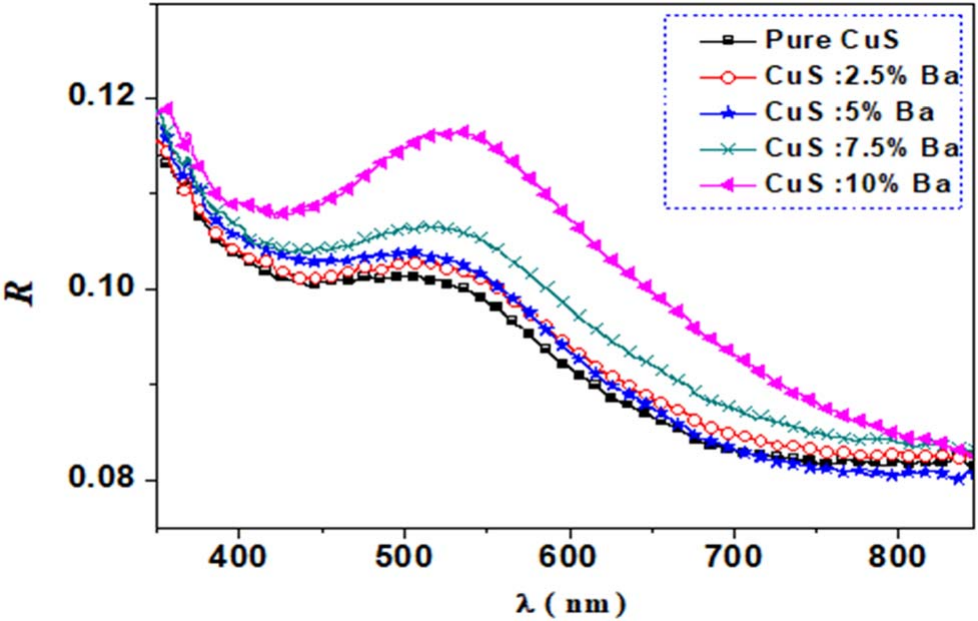
DR. spectra of pure and Ba-doped CuS nanoparticles.

The influence of Ba doping on the optical characteristics of CuS nanoparticles was investigated using diffuse reflectance spectrophotometer (DRS) spectra for produced NPs, as shown in Fig. 5. The diffuse reflectance spectrum in the range of 350–850 nm was recorded. One can see that there is a decrease in reflectance with the increase in wavelength. It is obvious to see that adding doping and raising its concentration in CuS NPs caused the reflectance values to rise.

#### 3.4.2 *F*(*R*)

**Fig. 6:**
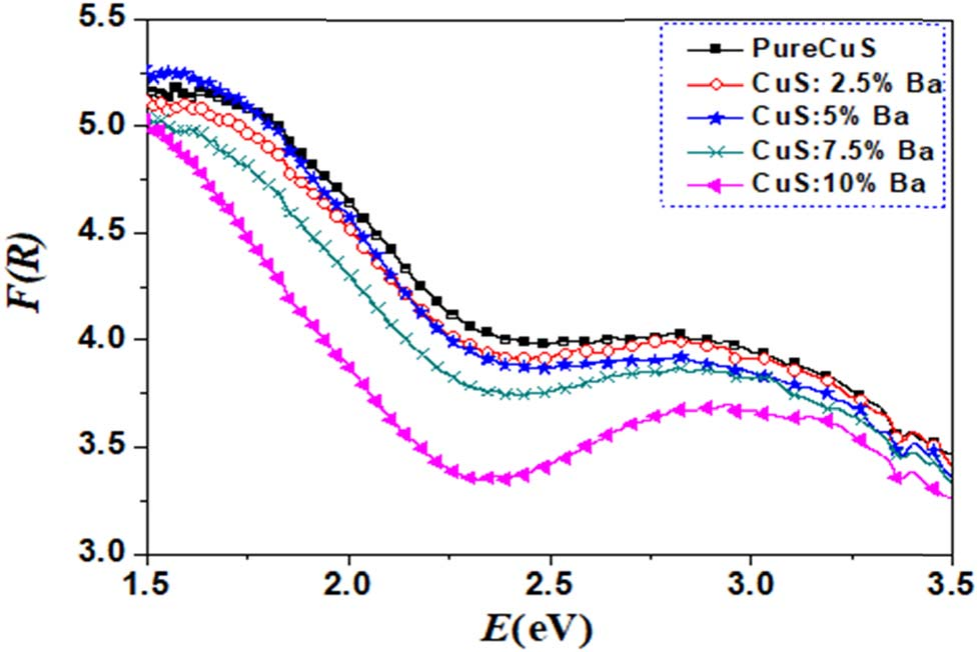
Absorption coefficient of pure and Ba doped CuS nanoparticles.

The band gap energy of the prepared samples was calculated using the Kubelka–Munk hypothesis. The Kubelka–Munk function *F*(*R*) of all samples was initially calculated using the reflectance data as follows:^25^5
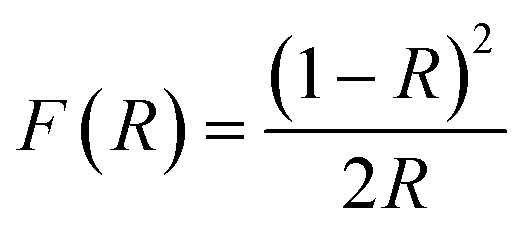
where *R* is the reflectance at each wavelength. According to Kubelka–Munk theory, *F*(*R*) is proportional to the absorption coefficient, and the absorption coefficient was determined as follows: *α* = *F*(*R*). Fig. 6 displays the obtained *F*(*R*) as a function of wavelength. In comparison to pure CuS NPs, samples that were doped with Ba have a lower absorption coefficient. In contrast to pure CuS NPs, a sample containing 5% Ba displayed a higher absorption coefficient. A 10% Ba sample was found to have the lowest absorption coefficient.

#### 3.4.3 Optical band gap energy (*E*_g_)

The optical band gap is an important characteristic needed for optoelectronic device design. It is commonly understood that optical transitions in semiconductor materials occur *via* direct and indirect transitions. The optical band gap *E*_g_ can be calculated using the fundamental absorption, which relates to electron excitation from the valence band to the conduction band.^26^ Using Tauc's equation, the energy gap is computed:^27^

**Fig. 7:**
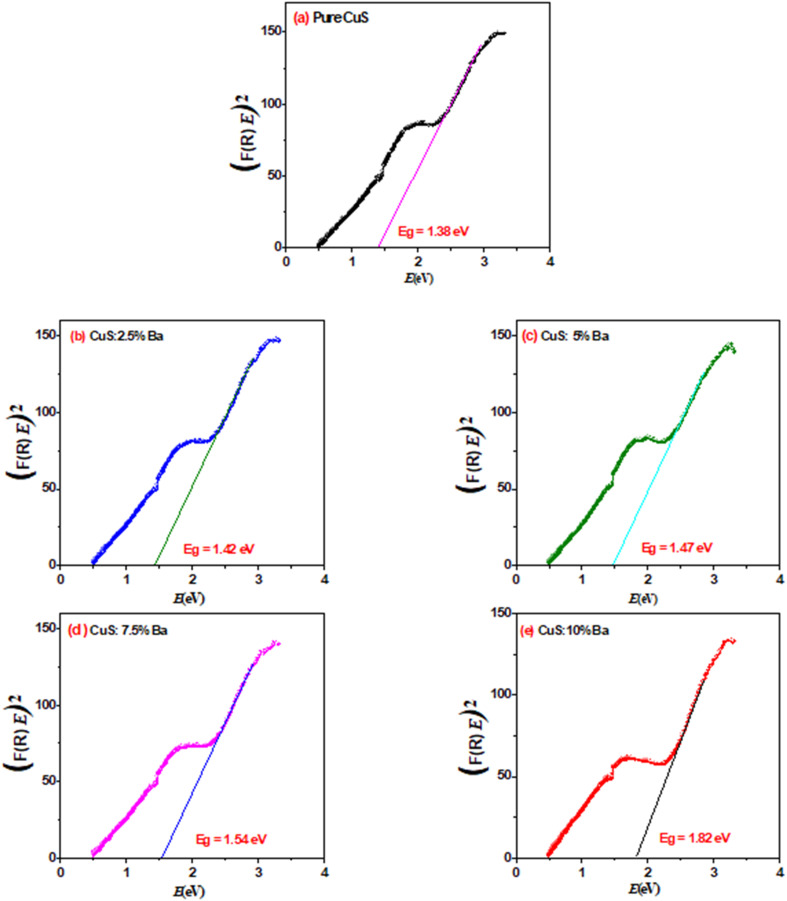
Plots of (*F*(*R*)*E*)^2^*vs.* photon energy (*E*) for pure and Ba doped CuS nanoparticles.

6 (*F*(*R*)*E*) = (*E* − *E*_g_)^*n*^

where *E* is the incident photon energy, *A* is a constant, *E*_g_ is the band gap energy of CuS, and *n* equals 1/2 for the allowed direct transition. The relationship between (*F*(*R*)*E*)^2^ and photon energy (*E*) is depicted in Fig. 7. The linear parts of these data were extrapolated to the *x*-axis (photon energy), and from there, the optical band gap values for pure and Ba-doped CuS were determined. Table 3 shows the obtained band gap *E*_g_ values for CuS and Ba-doped CuS NPs. As more Ba dopants are incorporated into the host matrix (CuS), it is clear that the optical band gaps of prepared samples increase. The 10% Ba-doped concentration shows the highest *E*_g_ value (1.82 eV). Comparing the *E*_g_ value of the pure CuS NPs to the bulk CuS value (*E*_g_ = 1.85 eV), it was found that the pure CuS NPs have a low *E*_g_ value of 1.38 eV.^6^ According to the literature, CuS nanoparticles with a band gap of 1.64 eV were produced by Saranya *et al.*^28^ and obtained by Aziz *et al.*^29^ with a band gap of approximately 1.29 eV. Furthermore, Pal *et al.*^30^ succeeded in producing CuS nanoparticles with a band gap of 1.09 eV. The results demonstrate that because doping provides free electrons, the band gap of doped CuS has a higher value than that of pure CuS, causing the Fermi level to split in the direction of the conduction band. This indicates that the blue shift, which is appropriate for optoelectronic devices, is shown in our results.^30^

**Table 3:** *E*_g_ of pure and Ba-doped CuS nanoparticles

Sample	*E*_g_ (eV)
Pure	1.38
2.5% Ba	1.42
5% Ba	1.47
7.5% Ba	1.54
10% Ba	1.82

#### 3.4.4 Extinction coefficient (*K*) and refractive index (*n*)

**Fig. 8:**
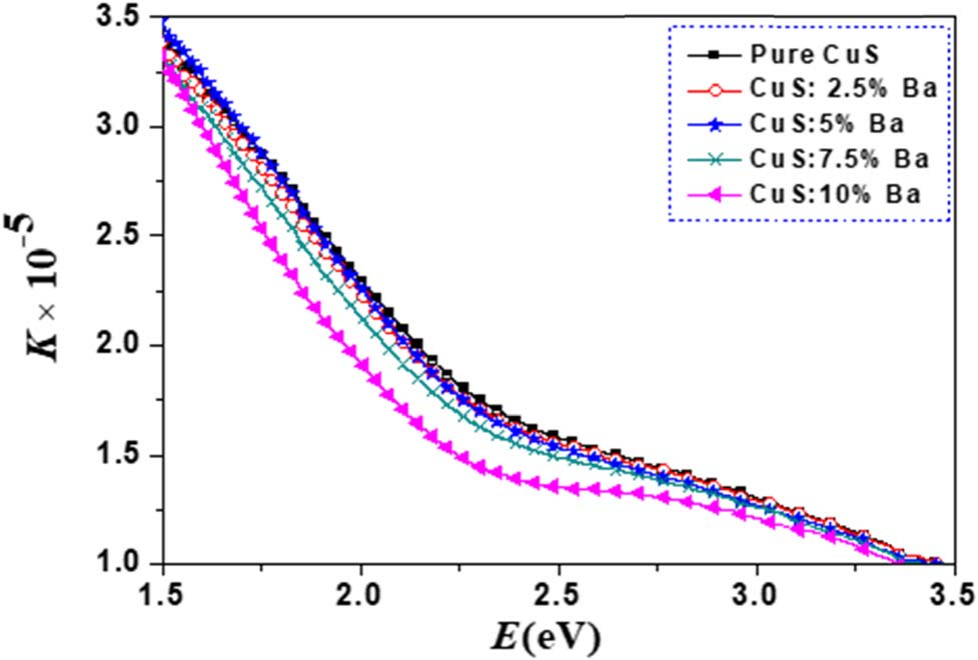
Extinction coefficient (*K*) *vs.* photon energy (*E*) for all of the samples.

**Fig. 9:**
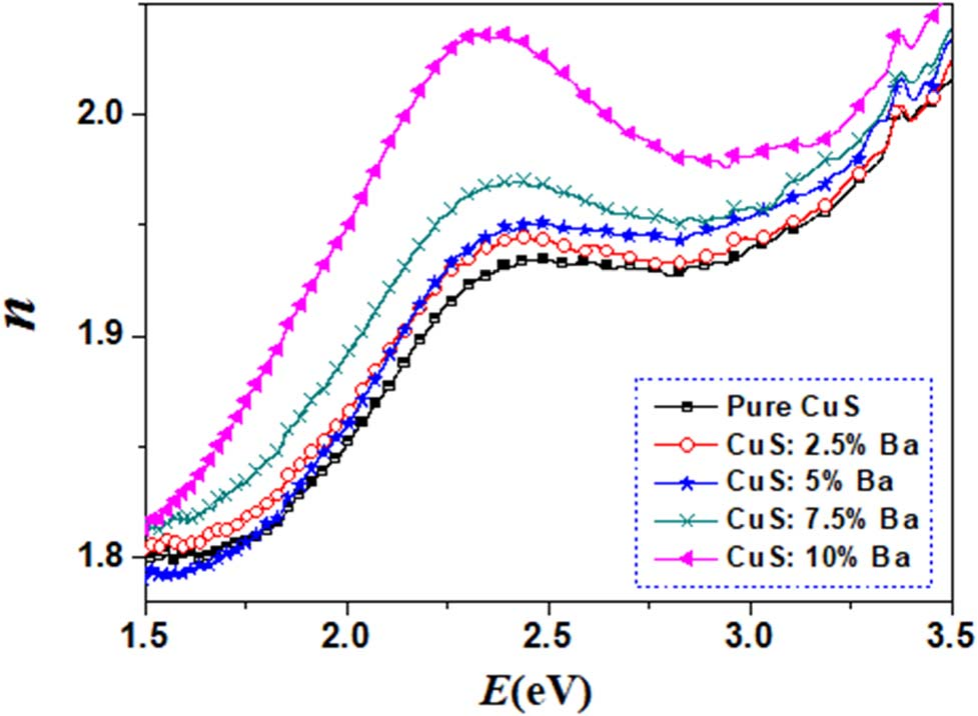
Refractive index (*n*) *vs.* photon energy (*E*) for all of the samples.

The extinction coefficient is a characteristic of a substance that indicates how strongly it absorbs light of a given wavelength. For applications in integrated optical devices, the refractive index of optical materials must be analyzed, and the refractive index of the material is also a critical parameter for device design. On the other hand, because of its interaction with the local field and the electronic polarization of ions within the material, the refractive index (*n*) is thought to be one of the most important characteristics of optical materials.^31^ These equations can be used to determine the extinction coefficient (*K*) and refractive index (*n*) of Cu_1−*x*_Ba_*x*_S nanoparticles:^32^7
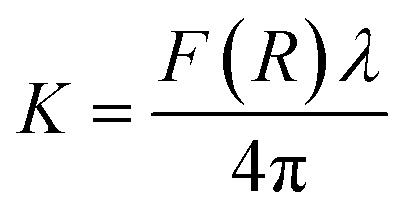
8
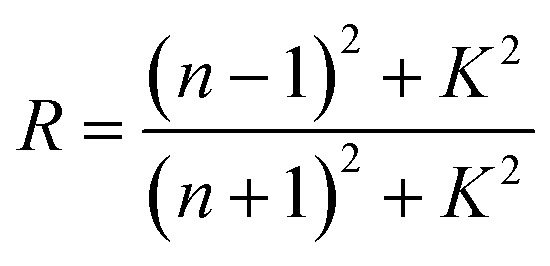
Since the value of *K*^2^ in eqn (8) is very small for dispersed reflectance, eqn (9) can be expressed as:9a
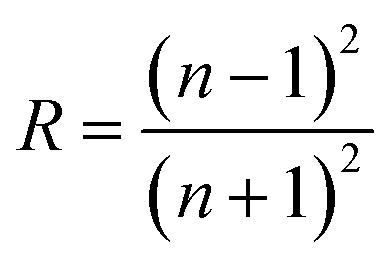
9b
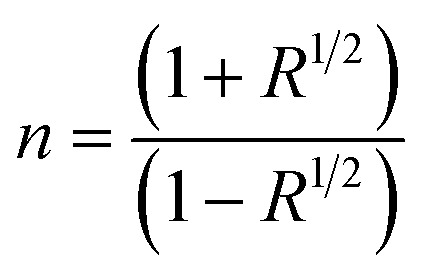
Fig. 8 and 9 show, respectively, how the extinction coefficient (*K*) and refractive index (*n*) vary with incident photon energy (*E*). It has been found that the extinction coefficient generally decreases as photon energy rises. With the exception of the 5% Ba-doped sample, the values of the extinction coefficient (*K*) for Ba-doped nanoparticles are lower than those for pure CuS samples.

From Fig. 9, a trend in the change of the refractive index of the samples can be determined. It is clear that the refractive index increases with an increase in the photon energy (*E*), after which there is a decrease in higher photon energy. Additionally, as the amount of Ba dopants rises, the refractive index value does as well. Pure and Ba-doped CuS nanoparticles exhibit an increase in refractive index with increasing *E* (normal dispersion). Anomalous dispersion is the opposite type.^26^

### 3.5 Photoluminescence study

**Fig. 10:**
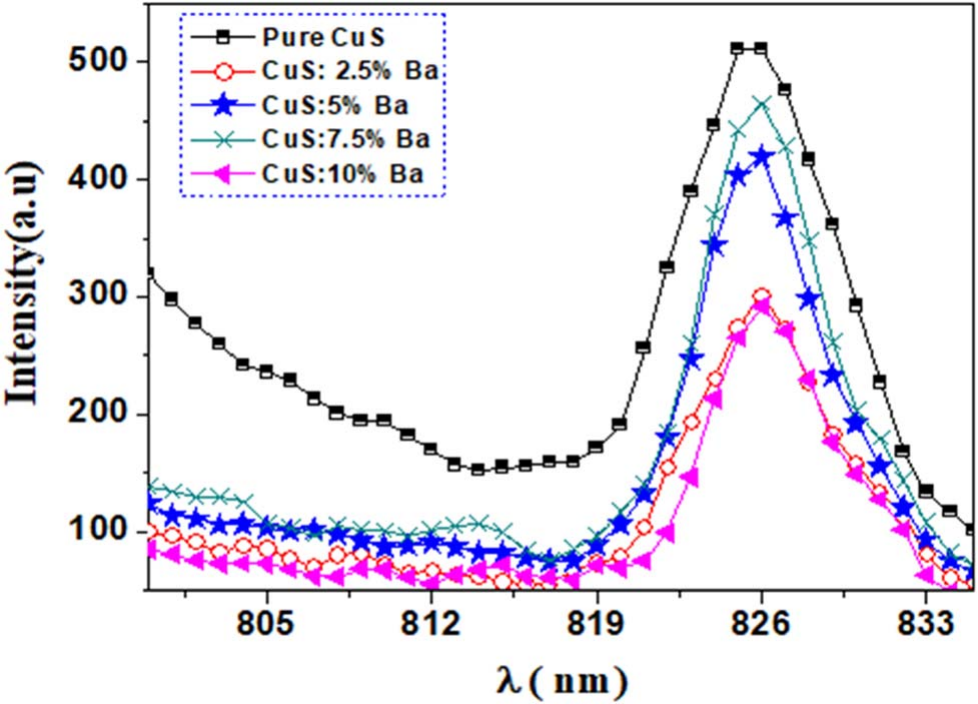
RT PL emission spectra at a *λ*_exc_ = 535 nm for pure and Ba doped CuS nanoparticles.

Fig. 10 shows photoluminescence (PL) spectra of pure and barium-doped copper sulfide (2.5%, 5%, 7.5%, and 10%) at room temperature. The measurements were performed at a wavelength of excitation of 535 nm. The spectra have a peak at 826 nm (*E* = 1.50 eV), observed from pure CuS and doped CuS nanoparticles. It is clear from Fig. 10 that pure CuS nanoparticles exhibit the maximum intensity of luminescence. The position of the band remains the same in the current study when Ba is added to the CuS matrix, but the intensity of the band changes in relation to the Ba concentration. A significant reduction in luminescence intensity is seen with the addition of Ba to the CuS host lattice. The reduced rate of electron and hole recombination causes the luminescence intensity to decrease, especially at 2.5 and 10% Ba doping concentrations. More photocatalytic activity in semiconductors is produced by lower recombination, which reduces photoluminescence intensity.^33,34^ The reduction of electron–hole recombination could therefore increase the photocatalytic activity of Ba-doped CuS.

The decrease in semiconductor PL emission band intensities is inversely related to the rate of radiative recombination.^35^ The four state possibilities of electron–hole pairs in polycrystalline semiconductors with a high hole conductivity (p-type) include: (1) band-to-tail (BT), in which a free electron is displaced in the valence band tail with a cavity; (2) band-to-band (BB), which consists of an electron and a hole that are both free, (3) a band-to-impurity (BI) state is one in which the acceptor state is not sufficiently deep to overlap with the tail of the valence band, and (4) donor–acceptor pairs consist of an acceptor state and a donor state that are deep enough to avoid overlapping with their respective band tails.^36^ In the current work, the photoluminescence band at 826 nm (1.5 eV) results from band-to-band in addition to band-to-impurity recombination in CuS.

### 3.6 Biological activity

#### 3.6.1 Anticancer activity

**Fig. 11:**
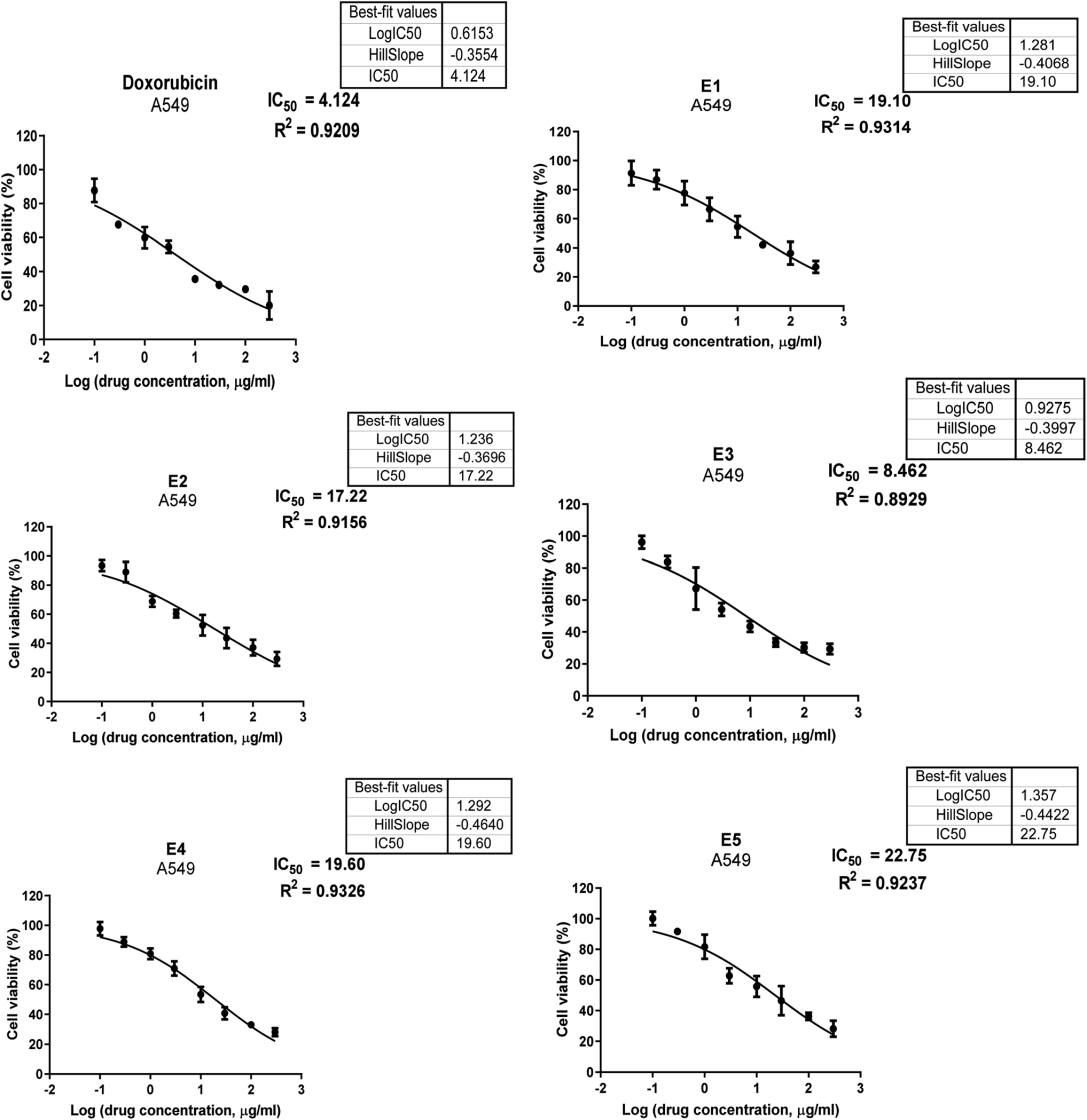
Cell viability % *versus* logarithm of the concentration and IC_50_ values obtained for tested samples.

**Fig. 12:**
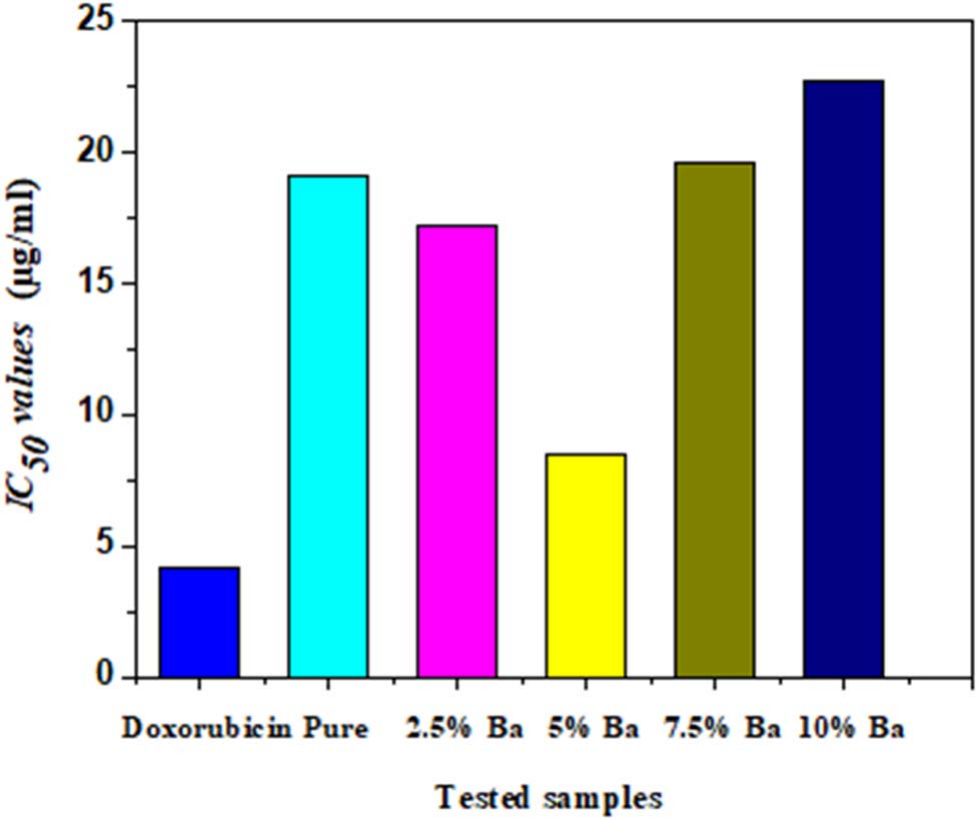
Comparison of the IC_50_ values of the tested samples against human lung cancer cell.

Using an MTT assay, the cytotoxicity of CuS NPs was examined. The samples were examined *in vitro* with human lung cancer cell lines (A549). Following a 72 hours treatment period, the effects of each sample on cell viability were measured at concentrations of 300, 100, 30, 10, 3, 1, 0.3, and 0.1 μg ml^−1^. Preliminary experiments were used to determine these concentrations and aiming to cover a broad spectrum from low to high concentrations. Fig. 11 displayed the cell viability data (obtained by the MTT experiment) *versus* logarithm of the concentration and IC_50_ values obtained for prepared samples as well as doxorubicin. Table 4 and Fig. 12 provide the cytotoxicity values (IC_50_) of the tested compounds in comparison to doxorubicin (a standard anticancer drug). The IC_50_ values are the doses in μg mL^−1^ that resulted in the death of 50% of tumor cells. The IC_50_ values relate to how well the sample inhibits the growth of cancer cells.^37^ As a result, the lowest concentration and IC_50_ values would be required for a highly effective cytotoxic drug. Overall, the results showed that the produced nanoparticles were very toxic to human lung cancer cells, particularly CuS with 5% Ba doping, which showed the most cytotoxicity against A549 cell lines with an IC_50_ of 8.46 μg ml^−1^, compared to the standard drug doxorubicin. Which recorded an IC_50_ value of 4.12 μg ml^−1^. Cytotoxicity first increases by up to 5%, then its value decreases when dopant concentration is increased.

**Table 4:** Cytotoxic activity of CuS and Ba-doped CuS nanoparticles against human lung (A549) cancer cell compared to doxorubicin

Samples	*In vitro* cytotoxicity (IC_50_) values^a^ (μg ml^−1^)
Doxorubicin	4.12
Pure	19.10
2.5% Ba	17.22
5% Ba	8.46
7.5% Ba	19.60
10% Ba	22.75

a IC_50_: inhibitory concentration (μg): 1–10 (very strong), 11–20 (strong), 21–50 (moderate), 51–100 (weak), and above 100 (non-cytotoxic).

These NPs easily penetrate the lung cells, producing ROS that induce cell death. The Ba–CuS NPs penetrate the cells and disrupt the function of the cancer cells. An important phenomenon is that materials with narrow energy band gaps absorb energy from sunlight and damage cancer cell membranes. The charge on the metal ions is another crucial characteristic for A549 cancer cell killing. It was shown that positive charges like Ba^2+^ and Cu^2+^ are produced by NPs to increase their effectiveness against cancer cells, and the cell membrane becomes negatively charged as a result of ionic interactions. The charge effect is responsible for both the position of attraction towards the negatively charged cell membrane and the attraction produced between the positive and negative charges. Cancerous cells die as a result of the aggressive interaction between Ba–CuS NPs and a surface with negative charges on cancer cell membranes.^38^

## 4. Conclusion

In the current study, we investigate how barium affects the structural, optical, and anticancer characteristics of copper sulfide (CuS) NPs prepared through a simple chemical co-precipitation technique. CuS-NPs were characterized using XRD and TEM to determine their structural characteristics. After doping by Ba, Scherrer's formula revealed an increase in crystallite size for the particles. With changing barium concentrations in CuS, the energy band gap shifted from 1.38 to 1.82 eV. The value of the refractive index of each sample was determined using the optical reflectance method, and the results show that the refractive index increases with increasing Ba dopants. Ba-doped CuS NPs have a positive impact on tumor toxicity in the A549 cancer cell line, suggesting that it may be an effective cancer treatment.

## Data availability

All data supporting the findings of this study are included in the manuscript.

## Conflicts of interest

There are no conflicts to declare.
